# Association between the Triglyceride-Glucose Index and the Risk of Large Artery Atherosclerotic Stroke

**DOI:** 10.1155/2022/5191581

**Published:** 2022-10-11

**Authors:** Mingfei Jiang, Huan Wu, Huiping Zhang, Fan Su, Lei Cao, Xia Ren, Grace Tatenda, Jian Hu, Mingjia Cheng, Yufeng Wen

**Affiliations:** ^1^Department of Diagnostics, Clinical School of Medicine, Wannan Medical College, Anhui, Wuhu 214002, China; ^2^Department of Prevention Medical, School of Laboratory Medicine, Wannan Medical College, Anhui, Wuhu 214002, China; ^3^Stroke Research Center, Department of Ultrasound, Ma'anshan People's Hospital, Anhui, Ma'anshan 243000, China; ^4^Department of Prevention Medical, School of Public Health, Wannan Medical College, Anhui, Wuhu 214002, China

## Abstract

The aim of this study is to evaluate the value of the triglyceride-glucose (TyG) index and the risk of large artery atherosclerotic (LAA) stroke. Information on general demographic and clinical characteristics, magnetic resonance angiography (MRA) examination, and blood biochemical index determination were obtained. Based on age stratification, three models to evaluate the odds ratio (OR) and the 95% confidence interval (95% CI) were employed to determine the correlation between the TyG index and the risk of LAA stroke. The most effective TyG index threshold in predicting a high risk of LAA stroke was identified using receiver operating characteristic (ROC) curve analysis. Logistic regression verified the association between the risk of LAA stroke and the TyG index. Both with and without age stratification, logistic regression analysis showed that the TyG index was a significant predictor of the occurrence of LAA stroke (*P* < 0.05). The maximum Youden index for determining a high risk of LAA stroke was found at a TyG index of 4.60. The area under the ROC curve was 0.69 (95% CI: 0.646–0.742, *P* < 0.05), sensitivity was 78.0%, and specificity was 63.4%. An elevated TyG index was remarkably associated with a high risk of LAA stroke.

## 1. Introduction

In an aging society, the fatality and disability rates of stroke in the elderly are estimated to be more than 30%, which brings a huge economic pressure and spiritual burden [1, 2]. Due to the lack of professional neurologists and magnetic resonance angiography (MRA) examination facilities, most community hospitals cannot quickly and accurately assess the condition of elderly stroke patients [3]. Therefore, it is of great clinical significance to explore a simple, rapid, and economical indicator to evaluate stroke.

Insulin resistance (IR) is a main feature of metabolic disorders and can independently predict the occurrence of cardiovascular disease [4, 5]. The homeostasis model assessment of IR (HOMA-IR) combines fasting blood glucose (FBG) and insulin concentration, which is a validated and frequently used marker of IR. However, its application is limited in clinical practice due to the atypical measurement of serum insulin levels [6, 7]. The triglyceride-glucose (TyG) [8, 9] index based on FBG and fasting triglyceride (TG) levels was first reported as a valuable marker of IR in 2008 and is closely related to HOMA-IR.

Fasting glucose and TG are well-known risk factors for stroke recurrence [10, 11]. A recent study reported that the TyG index is also used as a comprehensive indicator of acute hyperglycemia and hypertriglyceridemia, which is associated with early neurological deterioration (END) and early recurrent ischemic lesions (ERILs) in patients with acute ischemic stroke [12].

Large artery atherosclerosis (LAA) stroke is mainly caused by atherosclerosis and thrombosis, which leads to narrowing or even occlusion of the lumen, causing focal acute cerebral insufficiency [13–16]. At present, there are few studies on the relationship between LAA stroke and the TyG index. In this study, we not only verified the association between the TyG index and LAA stroke but also explored the most effective threshold value of the TyG index for the prediction effect of LAA stroke.

## 2. Methods

### 2.1. Study Participants

This was a retrospective cross-sectional study. From November 2013 to October 2018, 2,836 patients with suspected LAA stroke who met the inclusion criteria were admitted to Ma'anshan People's Hospital. Subjects were divided into two groups according to magnetic resonance angiography (MRA) results: LAA stoke group (*n* = 458) and non-LAA stroke group (*n* = 2378). Inclusion criteria were as follows: first onset, less than 7 days from symptom onset to hospital admission; aged 60–80 years; patients with no history of carotid artery stenosis interventional therapy; and patients with no history of heart disease, serious infection, or cancer. Exclusion criteria were as follows: history of cerebrovascular disease; previous use of hypoglycemic and lipid-lowering drugs; patients with chronic kidney disease, hepatitis, and liver cirrhosis; and history of stroke. Ethical approval to report this case was obtained from the Medical Ethics Committee of Ma'anshan People's Hospital (No. 2014001). Verbal informed consent was obtained from the patients for their anonymized information to be published in this article.

### 2.2. Research Flowchart

The flowchart for this research study is given as follows:


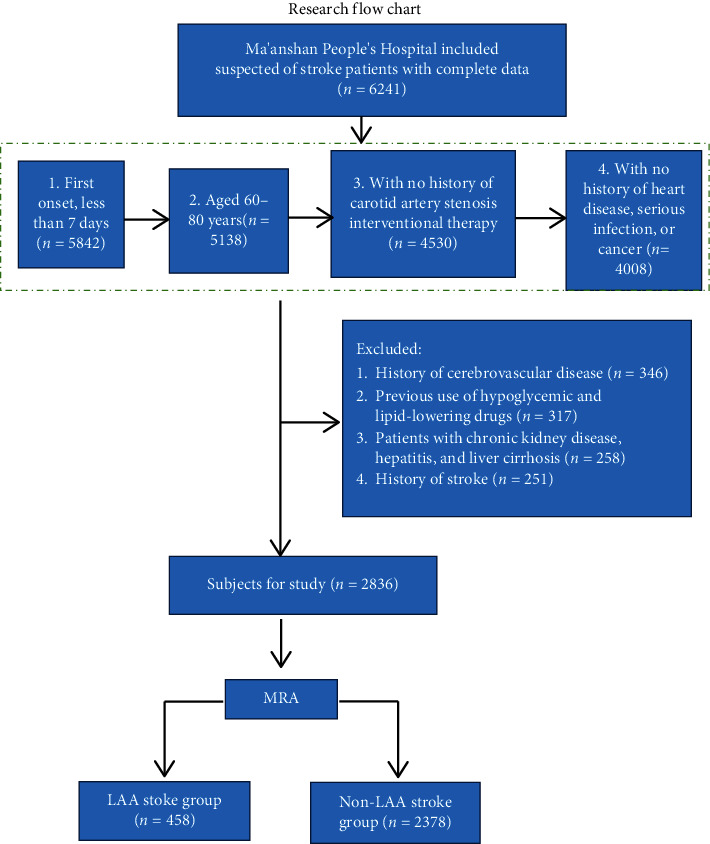


### 2.3. Clinical Data Collected

The recorded information included general demographic characteristics, clinical characteristics, MRA examination, and blood biochemical index determination. The questionnaire survey included baseline demographic characteristics (age, gender, height, and BMI) and classic risk factors (e.g., smoking and drinking). Face-to-face interviews with patients or family members were adopted. Clinical examinations included MRA examination, hypertension, and diabetes. Laboratory examinations included triglycerides (TG), total cholesterol (TC), high-density lipoprotein cholesterol (HDL_C), low-density lipoprotein cholesterol (LDL_C), and creatinine.

### 2.4. Indicators Calculated

TyG index was calculated as ln [fasting triglycerides (mg/dL) × fasting blood glucose (mg/dL)/2] [8, 9].

BMI was calculated as weight (Kg)/height square (m^2^) [17].

### 2.5. Diagnostic Criteria

LAA stroke was defined as initial MRA showing stenosis of 50% or more or occlusion due to intracranial or extracranial LAA and subsequent MRA showing persistent stenosis and occlusion and no cardioembolism and cryptogenic embolism with clinical stroke symptoms [12, 18].

Hypertension was diagnosed as systolic blood pressure values ≥ 140 mm Hg or diastolic blood pressure values ≥ 90 mm Hg or taking antihypertensive drugs.

Diabetes was defined as using antidiabetic drugs or FBG > 7.1 mmol/L or oral glucose tolerance test (OGTT) for 2 hours blood glucose > 11.1 mmol/L.

Dyslipidemia was defined as serum TC ≥ 5.2 mmol/L, LDL_C ≥ 3.38 mmol/L, HDL_C < 1.04 mmol/L, TG ≥ 1.7 mmol/L, or any lipid-lowering medication.

Smoking was defined as smoking more than one cigarette per day for 6 months.

Drinking was defined as alcohol intake exceeding 200 g per week for 1 year or longer.

### 2.6. Laboratory Assessment

A total of 5 mL of venous blood from each subject was used for the measurement of biochemical indices. The specimens were collected from the participants after 12 hours of fasting in the hospital and then were centrifuged and stored at −70°C until assayed. In Ma'anshan People's Hospital, an automatic biochemical analyzer (DPP-800, Roche, Germany) was used to determine serum TC, TG, LDL_C, HDL_C, and FBG.

### 2.7. MRA Measurement

All participants were examined with 3.0 T superconducting magnetic resonance imaging (GE, MR750) within 24 h of admission. First conventional plain scan: spin echo sequence T1WI horizontal axis, sagittal position, fast spin echo T2WI horizontal axis scan [19]; application of three-dimensional time of flight (3D-TOF) technology for data collection, the scan range was from below the foramen magnum to the top of the skull, and the scan line was parallel to the front and back joint line [20]. After scanning was completed, the obtained image data were transmitted to the processing workstation for maximum density projection processing. Three imaging physicians (more than 5 years of experience) in the hospital performed image evaluation to obtain imaging results.

### 2.8. Statistical Analysis

Statistical analysis was performed using SPSS 18.0. Continuous variables were expressed as mean ± standard deviation, and categorical variables were expressed as a percentage. The blood biochemical examination and physical index comparison between the LAA stroke group and the non-LAA stroke group were carried out using Student's *t*-test. Demographic characteristics, hypertension prevalence, diabetes prevalence, and dyslipidemia prevalence were analyzed using the chi-square test. In order to assess the association between the TyG index and LAA stroke, MedCalc software was used to determine the OR value and 95% CI with a logistic model under age stratification. Model I examined the bivariate association between the TyG index and LAA stroke. Model II was adjusted for hypertension, diabetes, smoking, and alcohol. Model III was further adjusted for SBP, FBG, TC, and TG. Receiver operating characteristic (ROC) curve analysis was used to estimate the accuracy of the TyG index to predict LAA stroke, and the Youden index analysis was used to estimate the most effective threshold value. Logistic regression was used to verify the association between LAA stroke and the TyG index stratified by the most effective threshold value, in which model I was unadjusted, model II was adjusted for dyslipidemia, diabetes, and gender, and model III was further adjusted for age, BMI, HDL_C, LDL_C, and DBP. A two-sided *P* < 0.05 was considered statistically significant.

## 3. Results

### 3.1. Demographic Characteristics and Clinical Parameters between LAA Stroke and Non-LAA Stroke

A total of 2,836 subjects were enrolled in this study, including 458 patients in the LAA stroke group and 2,378 patients in the non-LAA stroke group. The mean age of patients in the LAA stroke group was 69.84 ± 5.46 and in the non-LAA stroke group was 68.17 ± 5.52.  Table 1 shows there were no differences in BMI, dyslipidemia, UA, and CR between the LAA stroke and non-LAA stroke groups. The differences between the LAA stroke and non-LAA stroke groups with respect to the following parameters were all statistically significant (*P* < 0.05): smoking, drinking, hypertension, diabetes, and TyG index. The TyG index level of the LAA stroke group was higher than the non-LAA stroke group.

### 3.2. Multiple Logistic Analysis for LAA Stroke

Multiple logistic regression analysis was conducted to determine the most relevant factors predicting the occurrence of LAA stroke. Using LAA stroke as the dependent variable, patients with LAA stroke were recorded as “1” and those with non-LAA stroke as “0,” and age, smoking, drinking, hypertension, diabetes, SBP, TC, TG, FBG, and the TyG index were used as independent variables (SLS = 0.10, SLE = 0.05).  Table 2 shows that age, hypertension, and TyG index were significant predictors for the occurrence of LAA stroke.

### 3.3. Association between the TyG Index and LAA Stroke

**Figure 1:**
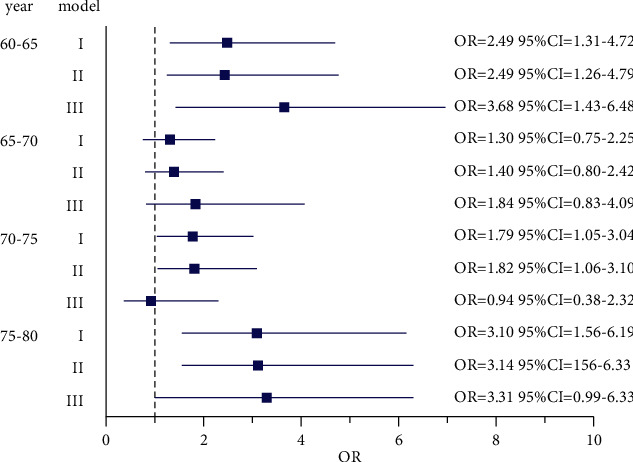
The relationship between the TyG index and LAA stroke determined using a logistic regression model. The dashed vertical line in the middle of the figure is invalid (odds ratio (OR) = 1), and each horizontal line is a connection between the upper and lower limits of the 95% confidence interval (CI). The length of the line segment intuitively represents the values of the 95% CI. The small squares are the OR and reflect the weight of the research. The model I was unadjusted; model II was adjusted for hypertension, diabetes, smoking, and drinking; and model III was adjusted for SBP, TC, TG, and FBG based on model II.

As shown in Figure 1, 60–65 years, the LAA stroke for the per-unit increase of TyG index was 2.49 in model I (OR: 2.49, 95% CI: 1.313–4.716); the adjusted LAA stroke for the per-unit increase of TyG index was 2.45 in model II (OR: 2.45, 95% CI: 1.256–4.788); the adjusted LAA stroke for the per-unit increase in TyG index was 3.68 in model III (OR: 3.68, 95% CI: 1.431–9.475); 65–70 years, the model showed no statistical significance between TyG index and LAA stroke. 70–75 and 75–80 years, LAA stroke was increased by 1.79 and 3.10 for the increased one unit in TyG index in model I (OR: 1.79, 95% CI: 1.047–3.041; OR: 3.10, 95% CI: 1.557–6.186). In model II, LAA stroke increased by 1.82 and 3.14 for each increase of TyG index by one unit (OR: 1.82, 95% CI: 1.063–3.096; OR: 3.14, 95% CI: 1.559–6.326).

### 3.4. ROC Curve of the TyG Index and LAA Stroke

**Figure 2:**
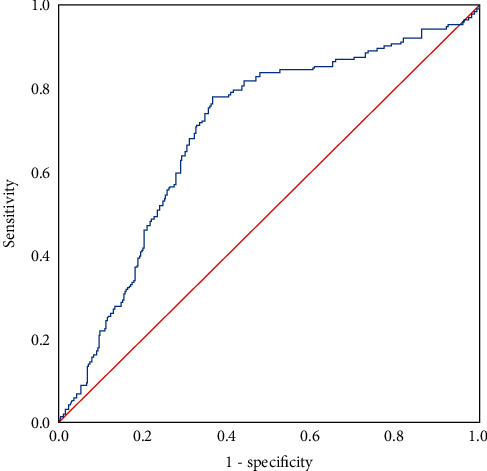
ROC curve for the TyG index as a predictor of LAA stroke.

Figure 2 shows the results of the ROC curve analysis: the TyG index was 4.60, which was the most effective threshold value for determining a high LAA stroke. The area under curve (AUC) was 0.69 (95% CI: 0.646–0.742, *P* < 0.05), sensitivity was 78.0%, and specificity was 63.4%.

### 3.5. Demographic Characteristics and Clinical Parameters of the TyG Index Stratified by the Most Effective Threshold Value

Taking the most effective threshold of the TyG index as the cutoff point, the TyG index was divided into a high TyG index (TyG > 4.60) group and a low TyG index (TyG ≤ 4.60) group.  Table 3 shows that hypertension, UA, and CR between patients with high TyG index and low TyG index showed no statistically significant difference. There were statistically significant differences in indicators such as age (*t* = 5.27, *P* < 0.001), gender (*χ*^2^ = 12.61, *P*=0.002), and dyslipidemia (*χ*^2^ = 79.49, *P* < 0.001). The LAA stroke was higher in patients with a high TyG index than in those with a low TyG index (*χ*^2^ = 12.82, *P* < 0.001).

### 3.6. Association between the TyG Index Stratified by the Most Effective Threshold Value and LAA Stroke

The results of the multivariable analysis of the association between the TyG index and LAA stroke are shown in Table 4, which includes ORs and 95% CI for high versus low TyG indices. In model I, a high TyG index was significantly associated with a high incidence of LAA stroke. The results remained highly consistent after adjusting for dyslipidemia, diabetes, and gender (model II). All adjusted variables in model II plus age, BMI, HDL_C, LDL_C, DBP, and FBG (model III), the TyG index remained significantly associated with LAA stroke incidence (*P* < 0.05). The ORs of high TyG index for LAA stroke were as follows: model I (OR: 1.44, 95% CI: 1.18–1.76); model II (OR: 1.33, 95% CI: 1.08–1.65); and model III (OR: 1.31, 95% CI: 1.04–1.65). The model I had the highest ORs for LAA stroke.

## 4. Discussion

In the present study, we found that the TyG index was positively correlated with high LAA stroke incidence. After adjusting for related risk factors, the association was observed to be significant. Stratified analysis based on the most effective threshold value, which was determined by ROC curve analysis, found that a high TyG index was significantly correlated with high LAA stroke incidence compared to a low TyG index. Continuous TyG index and stratified TyG index analysis indicated that it was a risk factor for LAA stroke.

IR is related to atherosclerosis through metabolic abnormalities [4, 5]. HOMA-IR, as a surrogate marker of liver insulin resistance, has been studied in the development of vascular plaque. The limitation of HOMA-IR is that insulin testing is a hard-to-reach and ubiquitous test, and its reproducibility is restricted [6, 7]. The TyG index, as a product of fasting blood glucose and TG, was closely related to glucose uptake stimulated by HOMA-IR and insulin [8]. A nationwide study in China reported that compared with other IR surrogate markers, the TyG index is more suitable for identifying individuals with vascular disease and metabolic abnormalities, with higher sensitivity and specificity [21]. Zhao et al. investigated the role of the TyG index in progressive diseases such as carotid atherosclerosis or peripheral artery disease [22]. Lu et al. provided evidence for a significant and positive correlation between the TyG index and subclinical atherosclerosis [8]. Nam et al. [12] and Irace et al. [23] reported that the increase in the TyG index accelerated the instability of atherosclerotic plaque. In addition, the TyG index is a predictor of hypertension prevalence in China and is a valuable indicator of obesity [24]. The TyG index is an appropriate diagnostic tool for IR, reflecting pathophysiological changes from a new perspective.

Due to the devastating effects of LAA stroke on human functionality and mortality, sensitive and specific diagnostic tools are indispensable to allow clinicians to provide rapid and effective care [25]. LAA stroke was caused by the narrowing or occlusion of the cerebral vessel lumen due to atherosclerosis and thrombus in the middle cerebral artery. Blood vessels in the brain with pathological changes, such as a rough inner wall and narrow lumen, form clumps, causing cerebral thrombosis [26]. The potential instability of atherosclerotic plaque may lead to arterial embolism, progressive progression from near occlusion to complete occlusion, and the disappearance of hemodynamics [27]. Zhao et al. showed that the TyG index is related to vascular injury [22]. Nam et al. indicated that a higher TyG index was associated with a higher incidence rate of subclinical cerebral small vessel disease [12]. Marfella et al. pointed out that the TyG index accelerated the instability of atherosclerotic plaque by affecting innate immune active substances and inflammatory active substances [28]. Carbonic anhydrase activation is associated with cardiomyopathy in diabetic subjects, indicating that carbonic anhydrase activation may mediate atherosclerotic cardiovascular disease [29]. Petrayevsky et al. found that the physiological and pharmacological levels of carbonic anhydrase activation were related to the TyG index [30].

Several studies were conducted to explore the most effective threshold value of the TyG index. A Mexican study found that the best threshold value of the TyG index for diagnosing type 2 diabetes was 4.65 [31]. A Chinese study pointed out that the TyG index of 4.37 in people with normal blood pressure accelerated arterial stiffness [32]. A Basel study reported that the best TyG index threshold value for stroke was 4.49 [33]. In this study, the best value of the TyG index determined using the ROC curve analysis for predicting LAA stroke was 4.60, which was equivalent to other studies.

Stroke prevention and treatment were listed as an important step of the Healthy China Campaign, and the first step in implementing the CSPPC project was to screen the population for stroke risk [34]. This paper verified the correlation between the TyG index and LAA stroke and further explored the most effective threshold of the TyG index, which had potential application value for community screening, accurate assessment, and clinical diagnosis of LAA stroke. This paper contributed to the development of the CSPPC project.

### 4.1. Study Limitations

Several limitations to this study should be acknowledged. First, it was a cross-sectional study. A causal relationship cannot be established directly based on the results. Indeed, prospective cohort studies are required to evaluate the predictive potential of the TyG index for the development of LAA stroke. Second, bias in the selection of the study population should be considered because we excluded patients who had previously used hypoglycemic and lipid-lowering drugs. Although this may lead to the above deviation, we decided to exclude the type and dose of administration that can directly affect the TyG index value. Third, after admission, we used the first data from the MRI examination to evaluate the LAA stroke results.

## 5. Conclusion

We found that a high TyG index is associated with an increased risk of LAA stroke. This suggested that the TyG index may be a useful marker for early identification of LAA stroke and can help to identify patients at high risk of cerebrovascular events, who benefit from early blood flow reconstruction. In addition, the TyG index deserves more attention in the treatment of cerebrovascular diseases. Further research is needed to determine whether reducing the TyG index can effectively reduce the incidence of cerebral infarction.

## Figures and Tables

**Table 1:** Comparison of demographic characteristics and clinical parameters between the LAA stroke and non-LAA stroke groups.

Variable	Non-LAA stroke (*n* = 2378)	LAA stroke (*n* = 458)	*t*/*χ*^2^	*P*
Age (years)	68.17 ± 5.52	69.84 ± 5.46	5.95	<0.001
60–65	758 (31.88)	91 (19.87)	35.73	<0.001
65–70	686 (28.85)	121 (26.42)		
70–75	529 (22.25)	138 (30.13)		
75–80	405 (17.03)	108 (23.58)		
Male (%)	1045 (43.94)	225 (49.13)	4.50	0.106
BMI (kg/m^2^)	23.95 ± 5.51	23.77 ± 3.23	0.92	0.356
Smoking (%)	602 (25.40)	145 (31.73)	8.64	<0.001
Drinking (%)	516 (21.77)	127 (27.85)	8.04	<0.001
Hypertension (%)	1525 (64.26)	369 (80.74)	47.21	<0.001
Dyslipidemia (%)	603 (25.49)	118 (25.88)	5.29	0.071
Diabetes (%)	1003 (42.32)	149 (32.60)	20.89	<0.001
SBP (mmHg)	134.8 ± 19.77	138.4 ± 16.97	4.04	<0.001
DBP (mmHg)	80.62 ± 30.77	80.73 ± 9.60	0.14	0.887
TC (mmol/L)	4.10 ± 1.04	4.28 ± 1.16	3.09	0.002
TG (mmol/L)	1.43 ± 0.32	1.63 ± 0.32	12.25	<0.001
HDL_C (mmol/L)	1.20 ± 0.30	1.19 ± 0.28	0.69	0.489
LDL_C (mmol/L)	2.27 ± 0.75	2.32 ± 0.90	1.18	0.265
TyG index	4.65 ± 0.34	4.75 ± 0.37	5.36	<0.001
UA (*μ*mol/L)	316.70 ± 99.34	318.70 ± 93.97	0.36	0.720
CR (*μ*mol/L)	78.18 ± 43.47	80.44 ± 39.39	1.00	0.316
FBG (mmol/L)	6.10 ± 2.79	6.61 ± 3.16	3.22	0.001

Data are presented as the mean ± SD or *n* (%); UA, uric acid; CR, creatinine.

**Table 2:** Multiple logistic analysis for LAA stroke.

Variable	*β*	SE	*χ * ^2^	*P*	OR	95% CI
Age (years)	0.04	0.01	20.81	<0.01	1.044	1.025–1.063
Hypertension	0.80	0.13	39.34	<0.01	2.230	1.736–2.865
TyG index	0.86	0.34	6.40	0.01	2.353	1.213–4.567

*β*, regression coefficient; SE, standard deviation; *χ*^2^, chi-square; OR, odds ratio; CI, confidence interval.

**Table 3:** Demographic characteristics and clinical parameters of the TyG index stratified by the most effective threshold value.

Variable	High TyG index (TyG > 4.6, *n* = 1137)	Low TyG index (TyG ≤ 4.60, *n* = 1699)	*t*/*χ*^2^	*P*
LAA stroke (%)	218 (19.17)	240 (14.13)	12.82	<0.001
Age (years)	69.11 ± 5.61	67.99 ± 5.46	5.27	<0.001
Male (%)	555 (48.81)	715 (42.08)	12.61	0.002
TC (mmol/L)	4.48 ± 1.20	3.91 ± 0.96	13.40	<0.001
TG (mmol/L)	2.08 ± 1.51	0.88 ± 0.24	26.57	<0.001
HDL_C (mmol/L)	1.13 ± 0.27	1.30 ± 0.32	15.24	<0.001
LDL_C (mmol/L)	2.41 ± 0.80	2.17 ± 0.96	7.22	<0.001
BMI (kg/m^2^)	24.42 ± 6.10	23.16 ± 3.32	6.36	<0.001
Hypertension (%)	1148 (67.65)	746 (65.84)	3.14	0.208
Dyslipidemia (%)	532 (31.50)	189 (16.68)	79.49	<0.001
Diabetes (%)	908 (53.45)	244 (21.57)	288.06	<0.001
SBP (mmHg)	135.90 ± 19.15	134.50 ± 19.78	1.88	0.060
DBP (mmHg)	82.23 ± 35.72	78.19 ± 10.32	3.54	<0.001
UA (*μ*mol/L)	317.40 ± 98.30	316.50 ± 98.89	0.24	0.812
CR (*μ*mol/L)	78.22 ± 37.50	79.03 ± 50.16	0.66	0.512
FBG (mmol/L)	7.57 ± 3.55	4.98 ± 1.11	23.83	<0.001

**Table 4:** Association between the TyG index stratified by the most effective threshold value and LAA stroke.

Model	*β*	S.E.	Wald *χ*^2^	*P*	OR	95% CI
I	0.37	0.10	12.73	<0.001	1.44	1.18–1.76
II	0.29	0.10	6.87	0.009	1.33	1.08–1.65
III	0.27	0.12	5.40	0.020	1.31	1.04–1.65

The model I was unadjusted; model II was adjusted for dyslipidemia, diabetes, and gender; and model III was adjusted for all variables in model II plus age, BMI, HDL_C, LDL_C, DBP, and FBG. *β*, regression coefficient; S.E., standard deviation; *χ*^2^, chi-square; OR, odds ratio; CI, confidence interval.

## Data Availability

No data were used to support this study.

## References

[1] Han J., Liu J., Wu Y. (2021). Long-term trends in the stroke prognosis among rural residents in China: a population-based surveillance study. *Risk Management and Healthcare Policy*.

[2] Saqqur M., Salam A., Ayyad A. (2020). The prevalence, mortality rate and functional outcome of intracerebral hemorrhage according to age sex and ethnic group in the state of Qatar. *Clinical Neurology and Neurosurgery*.

[3] Wu C. H., Chen S. T., Chen J. H. (2021). Diagnosis of extracranial carotid stenosis by MRA of the brain. *Scientific Reports*.

[4] Jeong S., Lee J. H. (2021). The verification of the reliability of a triglyceride-glucose index and its availability as an advanced tool. *Metabolomics*.

[5] Zhao Q., Cheng Y. J., Xu Y. K. (2021). Comparison of various insulin resistance surrogates on prognostic prediction and stratification following percutaneous coronary intervention in patients with and without type 2 diabetes mellitus. *Cardiovascular Diabetology*.

[6] Röhling M., Kempf K., Kolb H., Martin T., Schneider M., Martin S. (2021). The epidemiological boehringer ingelheim employee study (Part 3): association of elevated fasting insulin levels but not HOMA-IR with increased intima media thickness and arteriosclerosis in middle-aged persons. *Frontiers in Cardiovascular Medicine*.

[7] Lambie M., Bonomini M., Davies S. J., Accili D., Arduini A., Zammit V. (2021). Insulin resistance in cardiovascular disease, uremia, and peritoneal dialysis. *Trends in Endocrinology and Metabolism*.

[8] Lu Y. W., Chang C. C., Chou R. H. (2021). Gender difference in the association between TyG index and subclinical atherosclerosis: results from the I-Lan longitudinal aging study. *Cardiovascular Diabetology*.

[9] Simental-Mendía L. E., Rodríguez-Morán M., Guerrero-Romero F. (2008). The product of fasting glucose and triglycerides as surrogate for identifying insulin resistance in apparently healthy subjects. *Metabolic Syndrome and Related Disorders*.

[10] Shou J., Zhou L., Zhu S., Zhang X. (2015). Diabetes is an independent risk factor for stroke recurrence in stroke patients: a meta-analysis. *Journal of Stroke and Cerebrovascular Diseases*.

[11] Kwon H.-M., Lim J.-S., Park H.-K., Lee Y.-S. (2011). Hypertriglyceridemia as a possible predictor of early neurological deterioration in acute lacunar stroke. *Journal of the Neurological Sciences*.

[12] Nam K. W., Kwon H. M., Lee Y. S. (2021). High triglyceride-glucose index is associated with early recurrent ischemic lesion in acute ischemic stroke. *Scientific Reports*.

[13] Kim J., Shin S. J., Kang H. T. (2021). The association between triglyceride-glucose index, cardio-cerebrovascular diseases, and death in Korean adults: a retrospective study based on the NHIS-HEALS cohort. *PLoS One*.

[14] Liu H., Liu K., Pei L. (2021). Atherogenic index of plasma predicts outcomes in acute ischemic stroke. *Frontiers in Neurology*.

[15] Bergström G., Persson M., Adiels M. (2021). Prevalence of subclinical coronary artery atherosclerosis in the general population. *Circulation*.

[16] Ding Y., Xu Z., Pan Y. (2021). Association between CST3 gene polymorphisms and large-artery atherosclerotic stroke. *Frontiers in Neurology*.

[17] Lee J. W., Hong Y. M., Kim H. S. (2021). Identification of cardiovascular risk factors in obese adolescents with metabolic syndrome. *Frontiers in Pediatrics*.

[18] Yamashita S., Sato M., Yamazaki T., Yasuda S., Kato N. (2021). Identifying cerebral large vessel occlusion in acute ischemic stroke by MRI positioning scanning. *Neurologia Medico-Chirurgica*.

[19] Zhang D. F., Chen Y. C., Chen H. (2017). A high-resolution MRI study of relationship between remodeling patterns and ischemic stroke in patients with atherosclerotic middle cerebral artery stenosis. *Frontiers in Aging Neuroscience*.

[20] Li M. L., Lin Q. Q., Liu Y. T. (2020). The clinical value of head-neck joint high-resolution vessel wall imaging in ischemic stroke. *Journal of Stroke and Cerebrovascular Diseases*.

[21] Yu X., Wang L., Zhang W. (2019). Fasting triglycerides and glucose index is more suitable for the identification of metabolically unhealthy individuals in the Chinese adult population: a nationwide study. *J Diabetes Investig*.

[22] Zhao S., Yu S., Chi C. (2019). Association between macro- and microvascular damage and the triglyceride glucose index in community-dwelling elderly individuals: the Northern Shanghai Study. *Cardiovascular Diabetology*.

[23] Irace C., Carallo C., Scavelli F. B. (2013). Markers of insulin resistance and carotid atherosclerosis. A comparison of the homeostasis model assessment and triglyceride glucose index. *International Journal of Clinical Practice*.

[24] Xie F., Pei Y., Zhou Q., Cao D., Wang Y. (2021). Comparison of obesity-related indices for identifying nonalcoholic fatty liver disease: a population-based cross-sectional study in China. *Lipids in Health and Disease*.

[25] Kim S. W., Kim Y. D., Chang H. J. (2018). Different infarction patterns in patients with aortic atheroma compared to those with cardioembolism or large artery atherosclerosis. *Journal of Neurology*.

[26] Rosińska J., Ambrosius W., Maciejewska J., Narozny R., Kozubski W., Lukasik M. (2019). Association of platelet-derived microvesicles and their phenotypes with carotid atherosclerosis and recurrent vascular events in patients after ischemic stroke. *Thrombosis Research*.

[27] Jin G. (2017). The relationship between serum CXCL16 level and carotid vulnerable plaque in patients with ischemic stroke. *European Review for Medical and Pharmacological Sciences*.

[28] Marfella R., Paolisso P., Sardu C. (2021). SARS-COV-2 colonizes coronary thrombus and impairs heart microcirculation bed in asymptomatic SARS-CoV-2 positive subjects with acute myocardial infarction. *Critical Care*.

[29] Cho Y. R., Ann S. H., Won K. B. (2019). Association between insulin resistance, hyperglycemia, and coronary artery disease according to the presence of diabetes. *Scientific Reports*.

[30] Petrayevsky A. V., Trishkin K. S., Gndoyan I. A., Lomakina V., Adelshina N. (2021). Idiopathic intracranial hypertension (case study). *Vestnik Oftalmologii*.

[31] Almeda-Valdés P., Bello-Chavolla O. Y., Caballeros-Barragán C. R. (2018). Índices para la evaluación de la resistencia a la insulina en individuos mexicanos sin diabetes. *Gaceta Médica de México*.

[32] Wu Z., Zhou D., Liu Y. (2021). Association of TyG index and TG/HDL-C ratio with arterial stiffness progression in a non-normotensive population. *Cardiovascular Diabetology*.

[33] Calcaterra V., Verduci E., Schneider L. (2021). Sex-specific differences in the relationship between insulin resistance and adiposity indexes in children and adolescents with obesity. *Children*.

[34] Chao B. H., Yan F., Hua Y. (2021). Stroke prevention and control system in China: CSPPC-Stroke Program. *International Journal of Stroke*.

